# Real-World Evidence Evaluation of Respiratory Syncytial Virus (RSV) Vaccines: Deep Dive into Vaccine Adverse Events Reporting System

**DOI:** 10.3390/diseases14010029

**Published:** 2026-01-09

**Authors:** Thamir M. Alshammari, Mohammed K. Alshammari, Hind M. Alosaimi

**Affiliations:** 1 Department of Clinical Practice, College of Pharmacy, Jazan University, Jazan 45142, Saudi Arabia; 2 Pharmacy Practice Research Unit, College of Pharmacy, Jazan University, Jazan 45142, Saudi Arabia; 3 Department of Pharmaceutical Care Administration, Research and Studies Unit at Rafha General Hospital, Northern Border Healthcare Cluster, Rafha 76312, Saudi Arabia; mokaalshammari@moh.gov.sa; 4 Department of Pharmacy Services Administration, King Fahad Medical City, Riyadh Second Health Cluster, Riyadh 12211, Saudi Arabia; halosaimi@kfmc.med.sa

**Keywords:** Abrysvo, Arexvy, mRESVIA, pharmacovigilance, respiratory syncytial virus, RSV, VAERS

## Abstract

**Background**: Respiratory Syncytial Virus is a predominant source of morbidity and mortality, particularly among babies, the elderly, and immunocompromised patients. Recent developments in RSV vaccines, approved by the FDA for high-risk groups, have highlighted the necessity for post-marketing surveillance to evaluate their real-world safety and efficacy. **Method**: This study utilized data from the Vaccine Adverse Event Reporting System (VAERS) covering RSV vaccine administration between 2023 and May 2025. The VAERS database reported data on vaccine types, including Arexvy^®^, Abrysvo^®^, and mRESVIA^®^ was analyzed for adverse events and vaccination errors. The demographic information, vaccination trends, and hospitalizations post-vaccination among the vaccinated individuals were accessed. **Results**: The analysis revealed that the most common adverse events were mild, such as injection site pain, erythema, fatigue, and extremity pain. The data also showed a gradual increase in hospitalization rates from 4.8% in 2023 to 7.5% in 2025. Vaccination errors, including inappropriate administration during pregnancy and excess doses, were also observed. A notable trend was the growing proportion of patients who experienced no adverse events, with the highest rate of symptom-free reports seen in 2025 (25.9%). **Conclusions**: RSV vaccines demonstrate a generally acceptable safety profile based on post-marketing surveillance data. However, the observed increase in hospitalization rates, vaccination errors, and pregnancy-related outcomes warrants continued active surveillance and cautious interpretation.

## 1. Introduction

Respiratory syncytial virus (RSV) is a significant contributor to morbidity and mortality, affecting infants as well as the elderly [1]. The actual burden of RSV within the population may still be underestimated due to factors such as limited awareness and suboptimal diagnostic sensitivity in adults, the absence of comprehensive RSV surveillance systems, and the infrequent application of diagnostic testing [2,3]. Those at risk of severe RSV include premature infants, newborn children under the age of 2 years, pregnant women, immunocompromised individuals and adults above the age of 60 years [4,5]. Infants below the age of 6 months in low-and middle-income countries (LMICs) are reported to be more affected due to mortality and morbidity [6,7]. Respiratory syncytial virus (RSV) is a major contributor to hospital admissions for lower respiratory tract infections (LRTIs) in children under five globally, ranking as the second leading cause of infant mortality after malaria [7,8]. Worldwide, RSV accounts for an estimated 33 million LRTI cases annually that necessitate outpatient care, 3.6 million hospital admissions, and over 100,000 deaths, with more than 26,000 occurring in hospitals [8]. Even in developed countries, RSV leads to significantly higher death rates, being ten times more lethal than Influenza, with 3.1 deaths compared to 0.3 per 100,000 infants under one year old [9]. RSV is the most frequently identified cause of respiratory infections in young children, with nearly all children contracting it at least once by age two, often requiring medical intervention due to its clinical severity [7,8]. Research in the US and Europe indicates that the youngest children are more susceptible, and RSV infection places a greater clinical burden on infants up to 24 months old [10,11]; this may be partly because the lungs and immune system are still maturing during the early months of life [11,12].

In May 2023, the United States Food and Drug Administration (FDA) approved two new protein subunit RSV vaccines for adults above the age of 60 years: RSVPreF3 (Arexvy^®^, GSK, Rixensart, Belgium) [13] and RSVPreF (Abrysvo^®^, Pfizer, New York, NY, USA) [14]. The Centers for Disease Control and Prevention (CDC) recommended RSV vaccination on 21 June 2023, for adults aged 60 years and above, while in spring 2024, the vaccination was performed in 20–25% of the elderly, aged ≥ 60 years. Additionally, the FDA approved mRNA-1345 (mRESVIA^®^, Moderna, Cambridge, MA, USA) for RSV prevention in adults aged ≥ 60 years on 31 May 2024 [15]. To enhance the protection of infants, the FDA has approved the use of Abrysvo for pregnant women between 32 and 36 weeks of gestation to prevent RSV-related acute respiratory infections in newborns and infants [14]. The CDC and Advisory Committee on Immunization Practices (ACIP), in September 2023, recommended a single seasonal dose of Abrysvo^®^ vaccine for pregnant mothers between 32 and 36 weeks of gestation for prevention of RSV-associated acute respiratory infection in infants [16]. In order to prevent the infants from RSV-associated acute respiratory infection, the Abrysvo^®^ vaccine is administered to the pregnant mother, to provide the baby with immunity during the first 6 months after his birth [17]. The mother’s immunization with Abrysvo^®^ enables the fetus with passive immunity by transmitting the antibodies developed in the mother post-vaccination through the placenta [18]. The antibodies transferred to the fetus by passive transfer are very crucial for early protection of infants, thus enabling the infant’s immune response to fight against the RSV [19]. Alternatively, monoclonal antibodies, particularly Nirsevimab, represent a significant advancement in protecting infants from Respiratory Syncytial Virus (RSV). Unlike maternal vaccination, which relies on the transfer of antibodies during pregnancy, Nirsevimab is administered directly to newborns as a single intramuscular injection after birth [20]. This approach offers immediate and potentially extended protection throughout the RSV season due to its long half-life. Nirsevimab’s broader applicability makes it suitable for all infants, including those born prematurely or with health conditions that might hinder maternal vaccination [21]. As an additional measure to maternal vaccination, it provides an extra layer of defense against RSV. The use of Nirsevimab could reduce the burden on healthcare systems by preventing RSV infections and related hospitalizations [20,21]. Furthermore, it offers a potential solution for equitable protection, benefiting infants whose mothers may not have received or had access to maternal RSV vaccination [20]. The specific design of Nirsevimab to neutralize RSV may offer more targeted protection compared to naturally acquired maternal antibodies. This direct intervention strategy expands the range of tools available for combating RSV in infants, presenting a promising alternative or complement to maternal vaccination methods [20,21].

The availability of RSV vaccines in the United States (U.S.) provides an important public health measure to mitigate the burden of RSV disease among infants and older adults. Long-term complications associated with RSV have been noted in infants and young children. Early-life RSV has also been associated with subsequent long-term respiratory sequelae, including recurrent LRTD, recurrent wheezing, asthma, and lung function impairment, and these effects can persist into adulthood as chronic respiratory disease [22,23]. Given the clinical trial findings and the need to weigh the benefits against the risks for these newly approved products, vigilant post-marketing surveillance and close monitoring of reported adverse events following immunization (AEFI) have become indispensable [24,25]. There are some safety concerns regarding the safety of these vaccines, either from pre-approval studies or even after their approval. Key safety considerations for RSV vaccines within the United States include the potential for enhanced respiratory disease, allergic reactions, common side effects, and safety data in specific populations. Within the United States, regulatory agencies like the U.S. Food and Drug Administration (FDA) conduct thorough reviews of safety data before granting approval, and post-marketing surveillance systems (primarily the Vaccine Adverse Event Reporting System, or VAERS) are put in place to ensure continuous safety monitoring. These systems facilitate the detection of rare adverse events that may not have been evident in clinical trials due to limited sample sizes. Healthcare providers and vaccine recipients are encouraged to report any adverse events to these specific authorities (e.g., VAERS), contributing to the ongoing safety evaluation of RSV vaccines. This collaborative effort helps maintain public trust and ensures the continued safety and effectiveness of RSV vaccines in protecting the nation’s vulnerable populations, specifically infants, older adults, and pregnant women, from severe respiratory infections [26,27,28,29]. However, overall, the benefits of vaccination outweigh these potential adverse events [16].

Given the novelty of these vaccines, initial post-marketing safety data has begun to emerge from the first year of implementation (2023–2024). These preliminary analyses, often from passive surveillance systems, have provided early reassurance, largely confirming the safety profiles seen in clinical trials, such as injection site reactions, fatigue, and headache [30,31,32]. However, these initial studies possess significant limitations. Firstly, most reports cover a limited surveillance period, which is insufficient to capture potential rare or delayed-onset adverse events. Secondly, the real-world safety data is still scarce for key populations, particularly for pregnant women and subsequent infant outcomes following maternal immunization.

To address these gaps and guarantee the safety of RSV vaccines to the population administered with any of the RSV vaccines, the post-marketing surveillance is crucial to ensure vaccine safety due to its uncertainties. By analyzing the adverse event reports through the Vaccine Adverse Event Reporting System (VAERS), this study aimed to provide a more comprehensive, comparative analysis of real-world evidence on the safety profile of RSV vaccines. The findings are expected to have a pivotal role in guiding and making decisions for optimizing the immunization strategies by ensuring safety to infants, pregnant mothers, as well as the adult population who are prone to RSV-associated acute respiratory infection, irrespective of their age.

## 2. Methodology

### 2.1. Data Source

This was a retrospective pharmacovigilance observational study utilizing the VAERS database [33,34] during the period between January 2023 and May 2025. The VAERS database is jointly managed by the CDC and FDA, which was established in 1990 and is used to collect reports on post-vaccination adverse events reported by health care providers, patients, parents, and vaccine manufacturers, regardless of whether these events are directly or indirectly associated with the vaccine [35,36]. These data are available as freely accessed and ready to download as comma-separated value (CSV) files through this website (https://wonder.cdc.gov/wonder/help/vaers.html#VAERS%20Data%20Request; accessed on 19 July 2025).

The VAERS database included three datasets: (1) VAERSDATA, (2) VAERSVAX, and (3) VAERSSYPMTOMS. These datasets, a comprehensive data set includes information on demographic, medical history, specifics related to the adverse events and vaccine-related data. VAERSDATA, which is the largest file, provides information on patients’ demographic information, event outcomes, vaccination date, and onset date, and other information, while VAERSVAX dataset provides information related to vaccine name, manufacturer, route, site, and number of doses. The third file, which is VAERSSYMPTOMS, provides information on the symptoms, including up to 5 symptoms (i.e., symptom 1–symptom 5). All these datasets have shared one variable that is used to link this information, which is VAERS_ID [37]. Using the Medical Dictionary for Regulatory Activities (MedDRA), the adverse events are systematically coded in the database [37,38]. For signs, symptoms and diagnostic findings, each report is assigned multiple MedDRA Preferred Terms [37]. Our study followed the REporting of a Disproportionality Analysis for DrUg Safety Signal Detection Using Individual Case Safety Reports in Pharmacovigilance (READUS-PV) guidelines [39].

### 2.2. Eligibility Criteria

All reports of adverse events following immunization (AEFIs) were extracted from the VAERS database spanning from January 2023 to May 2025, to examine the safety profile of the RSV vaccines. Variables such as age, sex, symptoms (i.e., symptom 1–symptom 5), symptom text, vaccine type, vaccine name, onset of developing the AEFIs in days, site of administration, hospitalization, and vaccination date were analyzed in this study. It is worth mentioning that symptoms 1 to 5 were collected; however, duplications were removed.

All reported AEFIs were included in this study as events of interest; however, the exposures of interest were limited to RSV vaccines only.

### 2.3. Review of Reports

The raw data was accessed and downloaded from the VAERS website [34]. Using the methodology of Moro et al., utilizing MedDRA coding combined with text-string searches related to individuals who have received RSV vaccines and reported the adverse event or vaccination error to the VAERS [40]. Adverse events that are pregnancy-specific or non-pregnancy-specific adverse events were also selected for inclusion and reporting in this study after discussion with other co-investigators involved.

### 2.4. Ad Hoc Analyses and Review

For comparison purposes, especially with respect to AEFIs in pregnancy, analyses with the other two types of vaccines (Influenza virus vaccine and diphtheria, tetanus, and acellular pertussis vaccine, “Tdap”) were made. The selection of these vaccines was because these vaccines are used for kind of similar diseases system and can be used in pregnancy.

### 2.5. Statistical Analysis

Descriptive and inferential statistical analyses were conducted for both demographic and clinical characteristics of the variables studied in this research. All variables’ frequencies and percentages were calculated and presented.

A case/non-case pharmacovigilance disproportionality method was used to assess the signal of the top-reported AEFIs with the use of RSV vaccines. Using a contingency table, including all four situations of case/non-case pharmacovigilance method, which are as follows: (1) A as the reports of event of interest with the use of RSV vaccine, (2) B as the reports of other events with the RSV vaccine, (3) C as the reports of the event of interests with other vaccines, and (4) D as the reports with other events with other vaccines (Table 1) [41].

Both traditional and Bayesian disproportionality analyses were conducted to assess the signal of the top ADFIs with the use of RSV vaccines. Four data-mining metrics were used, including two traditional analyses (i.e., Reporting Odds Ratio (ROR), Proportional Reporting Ratio (PRR)) and two Bayesian analyses (i.e., Empirical Bayes Geometric Mean (EBGM), and Information Content (IC)) [42]. Using this dual method (i.e., Traditional and Bayesian analyses) was for the purpose of ensuring the reliability of the results of all four metrics for the signal of the event of interest with the studied vaccine. For the signal to be significant, ROR should be above 1, PRR above 2, EBGM should be equal to or more than 2, and any positive results of IC are considered a significant signal (Table 2) [43].

All statistical analyses were performed using R software (Version 4.2.2) and R Studio (Version 2024.04.2+764).

## 3. Results

A total of 6,569 patients were studied in our study. The patients studied were 2561, 3276, and 732 patients during 2023, 2024, and 2025, respectively. Table 3 highlights the age- and gender-wise distribution of patients who received RSV vaccines in the years 2023, 2024 and 2025. Results revealed that across all three years, a higher number of females received the vaccine compared to males. According to VAERS data, in 2023, a total of 1715 (67%) females were vaccinated compared to 647 (25.3%) males, with similar proportions observed in 2024 (2086, or 63.7% females vs. 784, or 23.9% males) and 2025 (459, or 62.7% females vs. 95, or 32.8% males). Findings further revealed that the majority of vaccine recipients were aged 60–75 years, comprising 46.6% in 2023, 37.9% in 2024, and 40.8% in 2025 as per the data available in VAERS.

Table 4 shows the frequency of RSV vaccine recipients in the U.S., including California, Florida, New York and Texas, reported the highest number of cases in 2023 and 2024, whereas 2025 (data until May 2025) shows the recipients in Florida, Georgia and California frequently received RSV vaccines.

Table 5 shows the distribution of intervals (in days) between the vaccination date and the onset of adverse events reported with the RSV vaccine during the study years (2023, 2024, and 2025). Results revealed that the majority of adverse events occurred on the same day as vaccination (0 days), accounting for 47.2% in 2023, 45.6% in 2024, and the highest in 2025 (54.6%). A significant portion of events occurred within 1–3 days post-vaccination, representing 42.1%, 28.5%, and 21.9% of reports in 2023, 2024, and 2025, respectively. Whereas the occurrence of adverse events beyond 3 days declined progressively across longer intervals, only a small percentage of adverse event reports were reported after 30 days. The findings further revealed that the occurrence of adverse events beyond 300 days was very rare. These findings suggest that most of the adverse events associated with RSV vaccination occurred shortly after administration.

The frequency of RSV vaccines administered based on brand names is presented in Table 6. The findings showed that in all three years, AREXVY was the most frequently administered vaccine, with 68.7% doses administered in 2023, 70.2% in 2024, and 68.9% in 2025. Likewise, ABRYSVO vaccine was administered to 30.5% patients in 2023, 28.3% in 2024, and 28.6% in 2025, whereas MRESVIA was administered to 21 patients in 2024, and 12 doses were administered in 2025.

**Figure 1:**
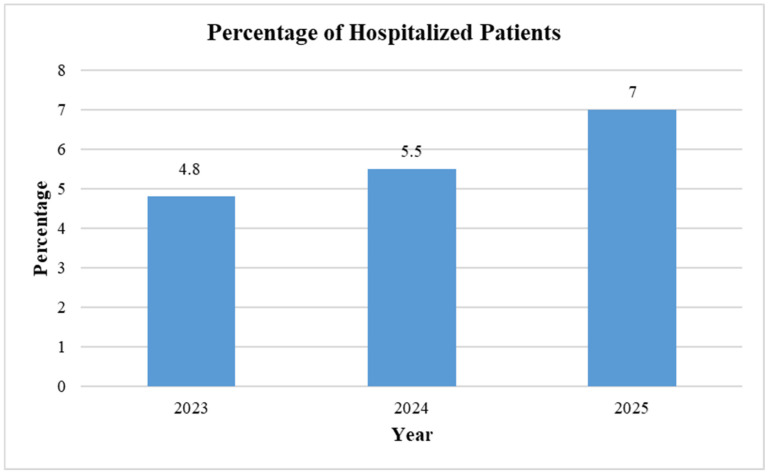
Percentage of patients hospitalized following RSV vaccine administration.

Figure 1 shows the percentage of patients hospitalized after RSV vaccine administration. The highest number of hospitalizations was reported in 2025 (7%), followed by 5.5% in 2024, and the least in 2023 (4.8%). The findings revealed a slight upward trend in the hospitalization rate after each passing year.

The frequency of RSV vaccine administration by the type of facility across all three years is presented in Table 7. The majority of vaccines were administered at pharmacies, peaking in 2023 at 63.2%, followed by 60.4% in 2025. Likewise, private clinics also saw steady use, increasing from 7.5% in 2023 to 9.3% in 2024 and 11.3% in 2025. The “Unknown” category remained substantial, especially in 2024 (32.4%), but dropped in 2025 (23.8%). Other facility types, such as military, public clinics, and nursing homes, contributed minimally throughout the period.

**Figure 2:**
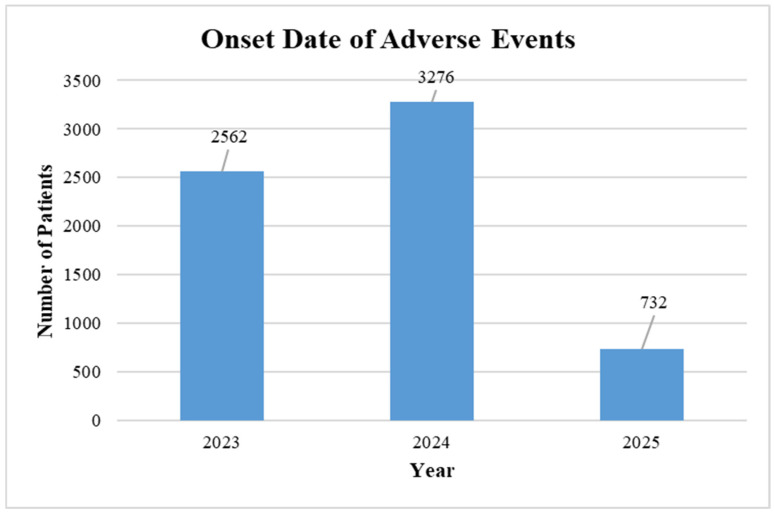
Onset date of adverse event symptoms associated with RSV vaccine.

Figure 2 highlights the date of onset of adverse events associated with vaccination across 2023, 2024 and 2025. The highest number of adverse events was reported in 2024, followed by 2023 and 2025.

Table 8 summarizes the frequency of symptoms reported following RSV vaccination across all three years. Injection site pain (11.2%) was the most commonly reported symptom, followed by pain (8.1%), injection site erythema (5.8%), and fatigue (4.8%). Whereas 8.1% of patients did not report any adverse events.

In 2023, the most reported symptoms following RSV vaccination were fatigue (11.6%), headache (11.1%), injection site pain (11.1%), pain in the extremities (11.1%), and general pain (10.5%), as shown in Table 9. In 4.3% of individuals, no adverse events were observed, indicating that the majority of patients experienced at least one symptom following vaccination as per the available data in the VAERS database.

In 2024, the most reported adverse event was injection site pain (12.5%), followed by injection site erythema (10.4%), injection site swelling (8.2%), and generalized pain (8.1%). In contrast, adverse events were not observed in 8.5% of patients, indicating a modest increase in the proportion of symptom-free cases compared to cases in 2023 (4.3%).

In 2025, findings revealed that injection site pain (5.9%) was the most commonly reported adverse event, followed by injection site erythema (5.3%) and pain in extremity (4.5%). Similarly, 19.7% individuals reported no adverse events following RSV administration, highlighting a substantial increase in symptom-free cases compared to cases in 2023 (4.3%) and 2024 (8.5%).

Table 10 summarizes the frequently reported vaccination errors related to RSV vaccine administration from 2023 to 2025. Findings revealed exposure during pregnancy as the most reported error in 2024 (10.9%), followed by 7.2% in 2025 and 4.3% in 2023. Likewise, extra doses being administered (36.2%) were the commonly reported error in 2025, followed by 15% in 2024 and 2.4% in 2023, indicating a significantly increasing trend from 2023 to 2025. Errors related to inappropriate age of product administration (5.1%) were the most frequently reported error in 2023, while the product use issues remained the most frequently reported vaccination error in 2025 after an extra dose was administered, and exposure during pregnancy errors.

### Pregnancy

The following results reflect pregnancy-related adverse events reported to the VAERS database. RSV vaccines administered during pregnancy have been associated with various safety concerns, as highlighted in several studies. Adverse events such as premature delivery and injection site reactions are the most common. However, other symptoms reported include headache, myalgia, nausea, and pre-eclampsia.

Table 11 underscores the frequency of symptoms reported in pregnant women in the VAERS database following administration of the RSV vaccine between 2023 and 2025. During the study years, premature delivery/labor remained the most frequently reported event, with the highest frequency in 2023 (18.6%), followed by 2024 (7.3%) and 2025 (13%). Injection site erythema was also commonly observed across the years, reported at 7% in 2023, 3.4% in 2024, and 4% in 2025. Cesarean section was recorded in 2023 (5.4%) and 2024 (2.0%), but not in 2025. Premature rupture of membranes occurred in 2023 (4.7%) and 2024 (1.4%), with no cases in 2025. Headache appeared only in 2024 (2.8%) and 2025 (4%). Diarrhea was noted in 2024 (1.4%) and increased to 4% in 2025. Stillbirth was reported minimally in 2024 (0.3%) but increased to 7% in 2025, while thrombocytopenia was only reported in 2025 (5%). All of these pregnancy-related reports were reported to VAERS.

Table 12 presents the disproportionality analysis of injection site pain, which consistently emerged as a safety signal associated with RSV vaccines across the years 2023 to 2025. Among the study years, injection site pain emerged as a strong safety signal in 2023 with high ROR and PRR values (3.80 and 3.74, respectively) and markedly elevated EBGM (3.40) and IC (1.77), indicating significantly higher reporting of pain site injection compared to other adverse events. In 2024, the disproportionality measures remained statistically significant but weaker (ROR 1.74, PRR 1.73, EBGM 1.64, IC 0.71), suggesting significantly higher reporting of injection site pain as a safety signal compared to other adverse events, though at a reduced magnitude vs. 2023. Similarly, in 2025, injection site pain associated with RSV administration emerged as a significantly higher reported adverse event compared with other events, but with lower magnitude as reported in the years 2023 and 2024 (ROR 1.69, PRR 1.67, EBGM 1.59, IC 0.67). This might be due to a smaller number of reports, as reporting only covered the period up to May 2025.

According to disproportionality analysis, pain emerged as a safety signal associated with RSV vaccines across the study years, as depicted in Table 13. Results indicate that in 2023, pain was significantly reported vs. other adverse events than expected following RSV immunization with ROR (1.81; 95% CI: 1.58–2.07), PRR (1.74; 95% CI: 1.52–2.00), EBGM (1.71), and IC (0.77), whereas, in 2024 and 2025 no significantly higher reporting of pain was observed following immunization.

## 4. Discussion

This pharmacovigilance descriptive safety study has used VAERS databases, which the US FDA and the CDC manage. Post-marketing surveillance data of RSV revealed that most reported adverse events were mild, with further insights expected from ongoing clinical trials to guide future guidelines. The overall profile of these RSV vaccines supports the vaccines’ critical role in mitigating RSV-related morbidity and mortality.

Our analysis found that the most commonly reported adverse events were local reactions, such as pain and redness at the injection site, and systemic events, including pain in the limbs, fatigue, and headaches. These results are consistent with those from similar studies [31,48,49]. For instance, V-safe data indicated that Arexvy^®^ recipients reported local (43.9%) and systemic (36.6%) symptoms more frequently than Abrysvo^®^ recipients (20.3% local, 21.6% systemic) [30], a trend our data also reflects in the volume of reports attributed to each product. The “no adverse event” category in VAERS, which peaked at 19.7% in 2025, must be interpreted with caution. As VAERS is a passive system, this figure reflects only an absence of reports and cannot be used to calculate a true rate of individuals with no adverse events; see the Supplementary Materials (Table S1).

Also, our study found that pregnancy-related adverse events reported for Tdap and Influenza vaccines provide valuable insights. The data indicates different patterns between the two vaccines. For instance, Tdap vaccines showed a higher percentage of “no adverse events,” but also highlighted specific fetal concerns like fetal deaths and abnormal heart rates, particularly in 2025. In contrast, high-risk pregnancies were more commonly reported with Influenza vaccines in the same year. Both vaccines noted hemorrhage and delivery issues, though categorized differently. However, these risks occurred in very few cases in this study; see the Supplementary Materials (Tables S3 and S6).

Our disproportionality analyses show a significant association between the signal of injection site pain and use of the RSV vaccine in 2023. However, this signal weakened in 2024 and 2025, and this trend may be due to the issue of underreporting commonly associated with spontaneous reporting systems, or perhaps a “reporting fatigue” after the initial novelty of the vaccines wore off. The mRNA-1345 RSV vaccine has been associated with side effects including headache, fatigue, myalgia, arthralgia and pain at the injection site [50]. This aligns with our findings, as reported by another study, the pain in extremity, headache, and fatigue were among the most frequently reported events among nonserious VAERS reports [30]. In this study, the adverse events of RSA vaccines reported in the VAERS database may indicate that most vaccine recipients were aged between 60 and 75 years. This demographic trend is expected, given that the US Advisory Committee on Immunization Practices’ (ACIP) recommendation focuses on adults aged 75 and older, and those aged 60–74 at higher risk [15]. The mRESVIA^®^ vaccine is still under post-marketing surveillance due to its recent approval. The RSV vaccination and management landscape, especially for infants, is not clear. There was a pattern of increase in hospitalization as it increased from 4.5, 5.5, and 6 in 2023, 2024, and until May 2025, respectively. However, although there is an increase in the pattern of hospitalization with RSV vaccine reports, there were no statistically significant signals in all four-disproportionality metrics; see the Supplementary Materials (Table S7).

Overall, this study found that there were more adverse event reports in females and individuals aged 60–75 years related to RSV vaccinations between 2023 and 2025. Among various states in the USA, California, Florida, New York, Georgia, and Tennessee reported the highest numbers of RSV vaccination adverse reports. The higher vaccination rates in Florida and California may be attributed to Florida having the second-largest population of citizens aged 60 and older, following California, as per the U.S. Census Bureau in 2023 [51]. It is critical to emphasize that our data reflects reporting activity, not vaccination rates.

Our research study identified premature delivery/labor, stillbirth, cesarean section, injection site erythema, thrombocytopenia, diarrhea, and positive RSV infection as the most reported among pregnant women from 2023 to 2025. These results align with other studies that have reported similar outcomes. A real-world pharmacovigilance study using the VAERS database to assess the safety of RSV vaccines in pregnant women found preterm birth to be the most frequently and significantly reported adverse event [28]. The Food and Drug Administration (FDA) also reported 1% higher risk of preterm birth following RSV administration in the RSV group compared to the control group [52], and another phase-III trial reported a 1.9% higher risk [53]. However, a cohort study examining the safety of RSV vaccines in pregnant women when administered at the 34th week of pregnancy found no statistically significant increase in preterm births, suggesting that administration timing (within the 32–36 week ACIP recommendation) may be a critical factor [16]. Other reported pregnancy-related AEs in our study, such as stillbirth and cesarean section, are also consistent with other VAERS analyses [28]. Importantly, findings from the phase-3 RSVpreF maternal vaccine trial reported 10 stillbirths among 3,698 vaccinated pregnant participants (0.27%), indicating that stillbirths were rare among vaccine recipients performed in 18 countries over four RSV seasons (two in the Northern Hemisphere and two in the Southern Hemisphere) [49]. When contextualized against general population data, the observed preterm birth rate of 18.6% with the RSV vaccine in our study exceeds the global baseline range reported by the World Health Organization, where preterm births account for between 4% and 16% [54]. Nevertheless, this observation warrants further post-marketing surveillance and controlled studies to clarify potential causal relationships. The risk of preterm birth was less with both the Influenza and tdap vaccines, as the percentage was 1.6% and 5.3%, respectively. However, these results should be interpreted carefully since the differences in the diseases for which these vaccines are used, since the RSV might more serious and perhaps the risk of bias occur here. Non-pregnancy-related adverse events frequently observed in our study include headache, injection site erythema, positive RSV infection, and diarrhoea are consistent with the existing literature, where injection site reactions [55], headache [50,56,57] and premature rupture of membranes [53] were reported following RSV vaccination. Pre-eclampsia [49], eczema, neonatal jaundice, gastroesophageal disease and asthma are the additional adverse events associated with the RSV vaccine in addition to preterm births [58].

The research underscores the significance of prioritizing vaccinations for older adults over 60, female patients, and maintaining vigilant monitoring in states with high disease prevalence, particularly Florida, California, and New York. It also emphasizes the necessity for readiness to manage any adverse events swiftly at vaccination centres, even though most adverse events observed in the study were minor and self-resolving. Additionally, this study highlights the need for strict compliance with vaccine administration guidelines and protocols, as well as the education of healthcare providers, due to the higher incidence of vaccination errors identified; see the Supplementary Materials (Table S2). According to the current study, pharmacies were the most frequently reported locations for administering the RSV vaccine. This aligns with the 2024 estimates from the Centres for Disease Control and Prevention, which indicated that pharmacies administered RSV vaccines to 81.7% of patients, while only 7.9% received them in physician clinics [6]. The common adverse events experienced by recipients of RSV vaccine in Australia from private market/pharmacies were local reactions (30%), most frequent were pain, swelling and redness at the injection site, followed by fatigue (21%), muscle ache (myalgia; 13%) and headache (12%) [59].

In the US, community pharmacy has become commonplace for vaccine administration [60]. As per CDC estimates in 2024, 81.7% of patients approached community pharmacies for vaccine administration [6]. However, there exist many challenges that need to be alleviated through continual medical education and training programs for positive outcomes. Moreover, it is crucial to develop more robust monitoring and reporting mechanisms to enable the prompt identification and management of negative reactions following vaccine administration. An extensive public education initiative should be rolled out to inform both healthcare providers and the public about the correct use of vaccines, their administration, contraindications, and the significance of reporting post-vaccination incidents to the VAERS database.

Retrieval of data from the VEARS database enabled the expansion of post-marketing surveillance of RSV vaccines approved by the FDA in 2023 to include real-world data on adverse events that were not reported in pre-marketing clinical trials. This study gives a holistic approach for these AEFIs associated with RSV vaccines among those who have received them. Additionally, the study provides a generalizable understanding of the RSV vaccine’s safety profile across different demographic groups.

However, this study has several limitations, since the database that was used was considered a passive reporting system, and the data source we used was from the VAERS database, so the relationship between the RSV vaccine and AEFI cannot be confirmed in this study (i.e., we cannot conclude causality). Also, we only have data for those who report AEFIs with these vaccines, but not all people who got the vaccines (i.e., the denominator data); therefore, the results should be interpreted with caution, considering the missing information. Furthermore, given the nature of this passive reporting system, there is still concern about underreporting and missing important AEFI information. Moreover, as VAERS is a passive reporting system, the ‘no adverse event’ category only reflects the absence of reports in the database and does not represent the true absence of adverse events among all vaccinated individuals. In addition, there might be a source of notoriety bias as a type of selection bias in reporting more cases of those who were affected by the vaccine. Lastly, the data presented in 2025 were incomplete at the time of analysis (January–May 2025), hence making comparisons with the full years of 2023 and 2024 less meaningful. Therefore, it is important to consider this when comparing the AEFIs among the different years because there might be differences in getting RSV from month to month.

## 5. Conclusions

RSV vaccines demonstrate a generally acceptable safety profile based on post-marketing surveillance data, with most adverse events being mild and self-limiting. However, the observed increase in hospitalization rates, vaccination errors, and pregnancy-related outcomes warrants continued active surveillance and cautious interpretation. Further controlled studies with denominator data are needed to establish definitive risk assessments.

## Figures and Tables

**Table 1:** Contingency table used to conduct the disproportionality analyses.

	Event of Interest	Other Events
**Exposure of interest**	a	b
**Non-exposure**	c	d

**Table 2:** Disproportionality analyses metrics and their significant criteria.

Measures	Formula	Criteria
**ROR**	ROR=[a∗db∗c] ${95CI\%=e}^{ln\left(ROR\right)\pm 1.96\sqrt{\frac{1}{a}+\frac{1}{b}+\frac{1}{c}+\frac{1}{b}}}$	95%CI/ > 1, N ≥ 2
**PRR**	PRR=[aa+bcc+d] ${95CI\%=e}^{ln\left(PRR\right)\pm 1.96\sqrt{\frac{1}{a}+\frac{1}{b}+\frac{1}{c}+\frac{1}{b}}}$	PRR ≥ 2, χ^2^ ≥ 4, N ≥ 3
**EBGM**	$\left[\frac{a\left(a+b+c+d\right)}{\left(a+c\right)\left(a+b\right)}\right]$ ${95CI\%=e}^{ln\left(EBGM\right)\pm 1.96\sqrt{\frac{1}{a}+\frac{1}{b}+\frac{1}{c}+\frac{1}{b}}}$	EBGM ≥ 2
**IC** **IC025** **IC975**	Log2 $\left[\frac{N\;observed+0.5}{N*expected+0.5}\right]$ IC − 3.3 × (O + 0.5) − 1/2 − 2 × (O + 0.5) − 3/2 IC + 2.4 × (O + 0.5) − 1/2 − 0.5 × (O + 0.5) − 3/2	Lower limit of 95% CI ≥0

CI, Confidence Interval; EBGM, Empirical Bayes Geometric Mean; IC, Information Component; PRR, Proportional Reporting Ratio; ROR, Reporting Odds Ratio. N ∗ expected = (Ndrug × Nreaction)/Ntotal. [44,45,46,47].

**Table 3:** Demographics of patients who received the RSV vaccine and experienced injection site pain in the VAERS database between 2023 and 2025.

Variables	2023		2024		2025	
	N	%	N	%	N	%
**Gender**						
Female	1715	67.0	2086	63.7	459	62.7
Male	647	25.3	784	23.9	218	29.8
Unknown *	199	7.8	406	12.4	55	7.5
**Age in Years**						
<18	26	1.0	56	1.7	15	2.0
≥18–59	185	7.2	279	8.5	50	6.8
≥60–75	1194	46.6	1240	37.9	299	40.8
≥76	604	23.6	759	23.2	223	30.5
NA	552	21.6	942	28.8	145	19.8
**Total**	**2561**		**3276**		**732**	
	**Injection Site Pain (by Sex)**					
**Gender**	**61**		**94**		**18**	
Female	37	60.7	63	67.0	15	83.3
Male	20	32.8	25	26.6	3	16.7
Unknown	4	6.6	6	6.4	0	0.0
**Age in Years**						
18–59 years	3	5.8	–	–	–	–
60–75 years	25	48.1	55	67.9	13	86.7
>75 years	24	46.2	26	32.1	2	13.3
**Total Injection Site Pain Reports**	**52**		**81**		**15**	

* Indicates VAERS reports where gender information was missing or not specified (neither male nor female); NA: not specified.

**Table 4:** Top five states in which patients received RSV Vaccine in 2023, 2024 and 2025.

State	2023		2024		2025	
	N	%	N	%	N	%
California	196	7.7	246	7.5	15	5.2
Florida	183	7.1	262	8	74	25.5
Georgia	–	–	–	–	31	10.7
New York	143	5.6	117	3.6	–	–
Pennsylvania	94	3.7	126	3.8	11	3.8
Tennessee	–	–	–	–	18	6.2
Texas	115	4.5	155	4.7	–	–

**Table 5:** Number of days adverse events following immunization as per the data years of 2023, 2024, and 2025.

Number of Days	2023		2024		2025	
	N	%	N	%	N	%
0	1208	47.2	1495	45.6	400	54.6
1–3	1078	42.1	934	28.5	160	21.9
4–7	203	7.9	201	6.14	30	4.1
8–14	117	4.6	117	3.57	38	5.2
15–30	80	3.1	77	2.35	25	3.4
31–60	20	0.8	32	0.98	11	1.5
61–100	0	0.0	6	0.18	2	0.3
101–300	0	0.0	10	0.31	5	0.7
>300	6	0.2	5	0.15	9	1.2
NA	556	21.7	926	28.3	175	23.9

NA: not specified.

**Table 6:** Frequency of RSV vaccine based on brand name within the VAERS database.

RSV Vaccine	2023		2024		2025	
	N	%	N	%	N	%
Abrysvo	780	30.5	927	28.3	209	28.6
Arexvy	1759	68.7	2300	70.2	504	68.9
MRESVIA	0	0	21	0.6	12	1.6
No Brand Name	22	0.9	28	0.9	7	1.0
Total	2561	100	3276	100	732	100

**Table 7:** Frequency of the type of facility administering the vaccine in 2023, 2024, and 2025.

Vaccination Center/Facility	2023		2024		2025	
	N	%	N	%	N	%
Military	10	0.4	11	0.3	3	0.4
Pharmacy	1619	63.2	1747	53.3	442	60.4
Public	19	0.7	34	1.0	7	1.0
Private	192	7.5	306	9.3	83	11.3
School or Student Health Clinic	1	0.0	1	0.0	0.0	0.0
Nursing Home or Senior Living Facility	10	0.4	41	1.3	4	0.5
Unknown	665	26.0	1060	32.4	174	23.8
Workplace Clinic	6	0.2	9	0.3	0	0.0
Other *	39	1.5	67	2.0	19	2.6

* Not specified in VAERS reports.

**Table 8:** Combined list of frequently reported symptoms following RSV vaccine administration in 2023, 2024 and 2025.

Symptom	N	%
Injection site pain	735	11.2
Pain	534	8.1
Injection site erythema	381	5.8
Fatigue	316	4.8
Pain in extremity	316	4.8
Headache	285	4.3
Injection site swelling	268	4.1
No adverse event	534	8.1

Percentage calculated in a total of 6569 patients who received RSV vaccine and reported an AEFIs in 2023–2025; no adverse event = refers to cases in which the administration of RSV vaccine does not result in any adverse event.

**Table 9:** List of frequently reported symptoms between 2023 and 2025 following RSV vaccine administration.

Symptom	2023		2024		2025	
	N	%	N	%	N	%
Fatigue	296	11.6	–	–	20	2.7
Headache	285	11.1	–	–	–	–
Injection site pain	284	11.1	408	12.5	43	5.9
Pain in extremity	283	11.1	–	–	33	4.5
Pain	269	10.5	265	8.1	–	–
No adverse event	110	4.3	280	8.5	144	19.7
Exposure during pregnancy	–	–	356	10.9	–	–
Injection site erythema	–	–	342	10.4	39	5.3
Injection site swelling	–	–	268	8.2	–	–
Arthralgia	–	–	–	–	20	2.7

Percentage calculated in a total of 2561, 3276, and 732 patients who received RSV vaccine in 2023, 2024, and 2025, respectively; no adverse event = refers to cases in which the administration of RSV vaccine does not result in any adverse event.

**Table 10:** List of frequently reported vaccination errors.

Vaccination Error	2023		2024		2025	
	N	%	N	%	N	%
Wrong product administered	68	2.7	201	6.1	11	1.5
Product administered to patients of inappropriate age	131	5.1	182	5.6	16	2.2
Incorrect dose administered	37	1.4	22	0.7	4	0.5
Syringe issue	20	0.8	61	1.9	3	0.4
Extra dose administered	61	2.4	490	15	265	36.2
Product use issue	81	3.2	204	6.2	16	2.2
Wrong technique in product usage process	11	0.4	0	0	0	0.0
Incorrect route of product administration	22	0.9	0	0	0	0.0
Underdose	33	1.3	0	0	0	0.0
Product preparation issue	48	1.9	0	0	4	0.5
Exposure during pregnancy	129	5	356	10.9	53	7.2

**Table 11:** Frequency of pregnancy-related symptoms observed in pregnant women in 2023, 2024, and 2025 within the VAERS databases.

Symptom	2023		2024		2025	
	N	%	N	%	N	%
Cesarean section	7	5.4	7	2.0	0	0
Diarrhea	0	0	5	1.4	2	4
Headache	0	0	10	2.8	1	4
Injection site erythema	7	7	12	3.4	2	4
Premature delivery/labor	24	18.6	26	7.3	7	13
Premature labor	–	–	–	–	0	–
Premature rupture of membranes	6	4.7	5	1.4	0	0
Stillbirth	0	0	1	0.3	4	7
Thrombocytopenia	0	0	0	0	3	5

Percentage calculated in a total of 129, 356, and 62 pregnant women exposed to RSV and reported AEFIs in 2023, 2024 and 2025, respectively; Respiratory Syncytial Virus infection refers to cases in which RSV infection was reported following vaccination. These reports do not imply that the vaccine caused RSV infection but represent events temporally associated with vaccination.

**Table 12:** Disproportionality analyses of injection site pain and the use of RSV vaccines in 2022 to 2025 using four data mining algorithms.

Year	Number of Injection Site Pains AEFIs *	Reported ADEs with the RSV Vaccines	ROR (95% CI)	PRR (95% CI)	EBGM (95% CI)	IC
2023	61	2934	3.80 (2.90–4.85)	3.74 (2.86–4.88)	3.40 (2.60–4.44)	1.77 (1.74–1.79)
2024	94	3615	1.74 (1.40–2.17)	1.73 (1.40–2.14)	1.64 (1.32–2.03)	0.71 (0.69–0.72)
2025 **	18	806	1.69 (1.04–2.77)	1.67 (1.03–2.74)	1.59 (1.00–2.60)	0.67 (0.56–0.73)

* Using Symptom1 variable; ** include data up to May 2025; CI: Confidence Interval; EBGM: Empirical Bayes Geometric Mean; IC: Information Component; PRR: Proportional Reporting Ratio; ROR: Reporting Odds Ratio.

**Table 13:** Disproportionality analyses of pain and the use of RSV vaccines during the period of 2022 to 2025 using four data mining algorithms.

Year	Number of Pains AEFIs *	Reported ADEs with the RSV Vaccines	ROR	PRR	EBGM	IC
			(95% CI)	(95% CI)		
2023	232	2934	1.81 (1.58–2.07)	1.74 (1.52–2.00)	1.71 (1.49–1.96)	0.77 (0.76–0.78)
2024	177	3615	1.01 (0.86–1.18)	1.01 (0.86–1.17)	1.01 (0.86–1.16)	0.007 (−0.002–0.014)
2025 **	33	806	0.86 (0.63–1.23)	0.86 (0.63–1.23)	0.87 (0.61–1.25)	−0.19 (−0.24–(−0.16))

* Using Symptom1 variable; ** include data up to May 2025; CI: Confidence Interval; EBGM: Empirical Bayes Geometric Mean; IC: Information Component; PRR: Proportional Reporting Ratio; ROR: Reporting Odds Ratio.

## Data Availability

The data are available with the corresponding author upon request.

## Supplementary Materials

The following supporting information can be downloaded at: https://www.mdpi.com/article/10.3390/diseases14010029/s1, Table S1: List of adverse events reported with influenza vaccines from 2023–2025; Table S2: List of vaccination errors reported with influenza vaccine from 2023-2025; Table S3: List of pregnancy related adverse events reported with influenza vaccines from 2023–2025; Table S4: List of adverse events reported with tdap from 2023–2025; Table S5: List of vaccination errors reported with tdap from 2023–2025; Table S6: List of pregnancy related symptoms reported with tdap from 2023–2025; Table S7: Pattern of hospitalization with RSV in 2023, 2024, and till May 2025.
