# Supplementary material for: Real-World Evidence Evaluation of Respiratory Syncytial Virus (RSV) Vaccines: Deep Dive into Vaccine Adverse Events Reporting System
Source: Diseases. 2026 Jan 9;14(1):29. doi: 10.3390/diseases14010029 (PMC12840037; doi:10.3390/diseases14010029)
Supplement: Supplementary file 1 [file diseases-14-00029-s001.zip › diseases-3911541-supplementary.pdf]

Supplementary Tables

Table-S1: List of adverse events reported with influenza vaccines from 2023-2025

**Symptom**	**2023**		**2024**		**2025**	
	**n**	**%**	**n**	**%**	**n**	**%**
No adverse event	22	35.5	21	42.9	6	42.9
Pain in extremity	4	6.5	-	-	-	-
Pain	3	4.8	-	-	1	7.1
Arthralgia	2	3.2	-	-	-	-
Chills	2	3.2	-	-	1	7.1
Headache	2	3.2	-	-	2	14.3
Abdominal pain	1	1.6	-	-	-	-
Bell's palsy	1	1.6	-	-	-	-
Disturbance in attention	1	1.6	-	-	-	-
Dizziness	1	1.6	3	6.1	-	-
Dyspnoea	1	1.6	3	6.1	-	-
Fatigue	-	-	5	10.2	-	-
Injection site pain	-	-	5	10.2	-	-
Cough	-	-	2	4.1	-	-
Delivery	-	-	2	4.1	1	7.1
Flushing	-	-	2	4.1	-	-
Injection site swelling	-	-	2	4.1	-	-
Anaphylactic reaction	-	-	1	2	-	-
Abdominal pain upper	-	-	-	-	1	7.1
Back pain	-	-	-	-	1	7.1
Immediate post-injection reaction	-	-	-	-	1	7.1
Pyrexia	-	-	-	-	1	7.1

Table-S2 : List of vaccination errors reported with influenza vaccine from 2023-2025

**Symptom**	**2023**		**2024**		**2025**	
	**n**	**%**	**n**	**%**	**n**	**%**
Exposure during pregnancy	37	59.7	31	63.3		
Exposure broaden during pregnancy	-	-	-	-	10	71.4
Product administered to patient of inappropriate age	10	16.1	3	6.1	1	7.1
Expired product administered	7	11.3	2	4.1	-	-
Extra dose administered	2	3.2	9	18.4	4	28.6
Wrong product administered	2	3.2	3	6.1	-	-
Maternal exposure before pregnancy	2	3.2	-	-	-	-
Accidental exposure to product	1	1.6	-	-	-	-
Product administered at inappropriate site	1	1.6	-	-	-	-
Product use issue	1	1.6	1	2	1	7.1
Inappropriate schedule of product administration	-	-	2	4.1	-	-
Maternal exposure during pregnancy	-	-	2	4.1	-	-

Underdose	-	-	1	2	-	-
Syringe issue	-	-	1	2	-	-
Product storage error	-	-	-	-	6	42.9
Incorrect dose administered	-	-	-	-	1	7.1

Table-S3 : List of pregnancy related adverse events reported with influenza vaccines from 2023-2025

Symptom	2023		2024		2025	
	n	%	n	%	n	%
Abortion spontaneous	2	3.2	1	2	1	7.1
Uterine dilation and curettage	1	1.6	2	4.1		
Caesarean section	1	1.6	-	-	-	-
Complication of pregnancy	1	1.6	-	-	-	-
Foetal cardiac arrest	1	1.6	-	-	-	-
Haemorrhage in pregnancy	1	1.6	-	-	1	7.1
Premature labour	1	1.6	-	-	-	-
Vaginal haemorrhage	1	1.6	-	-	-	-
Amniotic fluid volume decreased	-	-	1	2	-	-
Foetal heart rate abnormal	-	-	1	2	-	-
Ultrasound foetal abnormal	-	-	1	2	-	-
Delivery	-	-	-	-	1	7.1
High risk pregnancy	-	-	-	-	1	7.1
Pregnancy test positive	-	-	-	-	1	7.1
Pregnancy with contraceptive device	-	-	-	-	1	7.1

Table-S4: List of adverse events reported with tdap from 2023-2025

Symptom	2023		2024		2025	
	n	%	n	%	n	%
No adverse event	-	-	13	22.8	17	42.5
Dizziness	4	10.8	6	10.5	-	-
Fatigue	6	16.2	3	5.3	-	-
Sore Arm	6	16.2	-	-	-	-
Pain	3	5.4	-	-	-	-
Diarrhoea	3	8.1	2	3.5	-	-
Injection site pain	1	2.7	3	5.3	-	-
Headache	-	-	3	5.3	-	-
Chills	-	-	3	5.3	-	-
Dyspnoea	-	-	3	5.3	-	-
Injection site erythema	-	-	4	7	-	-
Palpitations	3	8.1	-	-	-	-
Pain in extremity	1	2.7	2	3.5	-	-

Anxiety	-	-	2	3.5	-	-
Erythema	-	-	2	3.5	1	2.5
Injection site pruritus	-	-	2	3.5	-	-
Nausea	1	2.7	-	-	-	-
Protein urine	1	2.7	-	-	-	-
Migraine	1	2.7	-	-	-	-
Pyelonephritis	1	2.7	-	-	-	-
Bell's palsy	1	2.7	-	-	-	-
Fasciitis	1	2.7	-	-	-	-
Arthralgia	-	-	-	-	1	2.5
Blood fibrinogen	-	-	-	-	1	2.5
Crossmatch	-	-	-	-	1	2.5
Disseminated intravascular coagulation	-	-	-	-	1	2.5
Haemorrhage	-	-	-	-	1	2.5
Immediate post-injection reaction	-	-	-	-	1	2.5
Limb discomfort	-	-	-	-	1	2.5
Loss of consciousness	-	-	-	-	1	2.5
Loss of personal independence in daily activities	-	-	-	-	1	2.5

Table-S5: List of vaccination errors reported with tdap from 2023-2025

**Symptom**	**2023**		**2024**		**2025**	
	**n**	**%**	**n**	**%**	**n**	**%**
Exposure during pregnancy	26	70.3	37	64.9	37	92.5
Extra dose administered	17	45.9	15	26.3	11	27.5
Product storage error	-	-	1	1.8	20	50
Wrong product administered	4	10.8	5	8.8	-	-
Inappropriate schedule of product administration	3	8.1	2	3.5	5	12.5
Product use issue	3	8.1	2	3.5	-	-
Incorrect route of product administration	-	-	2	3.5	-	-
Medication error	2	5.4	-	-	-	-
Wrong technique in product usage process	1	2.7	-	-	-	-
Underdose	1	2.7	-	-	-	-
Foetal exposure during pregnancy	-	-	1	1.8	-	-
Expired product administered	-	-	1	1.8	-	-
Product administered at inappropriate site	-	-	-	-	1	2.5

Table-S6 : List of pregnancy related symptoms reported with tdap from 2023-2025

Symptom	2023		2024		2025	
	n	%	n	%	n	%
No Adverse Events	14	37.8	-	-	-	-
Delivery	-	-	3	5.3	-	-
Premature delivery	-	-	3	5.3	-	-
Uterine contractions during pregnancy	2	5.4			-	-
Premature rupture of membranes	-	-	2	3.5	-	-
Uterine haemorrhage	1	2.7	-	-	-	-
Vaginal haemorrhage	1	2.7	-	-	-	-
Gestational diabetes	1	2.7	-	-	-	-
Cervical dilatation	-	-	1	1.8	-	-
Preterm premature rupture of membranes	-	-	1	1.8	-	-
Vaginal ulceration	-	-	1	1.8	-	-
Vulvovaginal pain	-	-	1	1.8	-	-
Vulvovaginal swelling	-	-	1	1.8	-	-
Foetal death	-	-	-	-	1	2.5
Foetal heart rate abnormal	-	-	-	-	1	2.5
Foetal monitoring abnormal	-	-	-	-	1	2.5

Table-S7: Pattern of hospitalization with RSV in 2023, 2024, and till May 2025

Year	Hospitalization	Reported ADEs with the RSV vaccines	ROR (95% CI)	PRR (95% CI)	EBGM (95% CI)	IC
2023	117	2934	0.23 (0.19-0.28)	0.26 (0.21-0.32)	0.27 (0.22- 0.32)	-1.90 (-1.91-(-1.88))
2024	171	3615	0.93(0.80-1.09)	0.93(0.80-1.09)	0.94 (0.80-1.09)	-0.09 (-0.10-(-0.08))
2025**	51	806	1.05 (0.78-1.41)	1.05 (0.78-1.41)	1.04 (0.78-1.40)	0.06 (0.03-0.08)
